# Calibrations, Validations, and Checks of a Dual 23 nm and 10 nm Diffusion Charger-Based Portable Emissions Measurement System (PEMS)

**DOI:** 10.3390/nano14151258

**Published:** 2024-07-27

**Authors:** Anastasios Melas, Maria Trikka, Sara Valentini, Giulio Cotogno, Barouch Giechaskiel

**Affiliations:** Joint Research Centre (JRC), European Commission, 21027 Ispra, Italy; anastasios.melas@ec.europa.eu (A.M.); maria.trikka@ec.europa.eu (M.T.); sara.valentini@ec.europa.eu (S.V.); giulio.cotogno@ec.europa.eu (G.C.)

**Keywords:** particle number, Euro 7, calibration, condensation particle counter (CPC), diffusion charger (DC), particle charge, measurement uncertainty, PEMS

## Abstract

The upcoming Euro 7 vehicle exhaust emissions regulation includes particle number (PN) limits for all vehicles, not only those with direct fuel injection. It also sets the lower detection particle size of the PN methodology to 10 nm from 23 nm. Recently, a commercial diffusion charger-based PEMS added the possibility of switching the lower size between 23 nm and 10 nm. In this study, we assessed the dual PEMS in the calibration laboratory using diffusion flame soot or spark discharge graphite particles following the regulated procedures. Furthermore, we compared the dual PEMS with a laboratory grade system (LABS) using soot, graphite, and vehicle exhaust particles. To put the results into perspective, we added comparisons (validations) of two additional 23 nm PEMSs with LABSs over a three-year period. The results showed that the differences of the 23 nm PEMSs remained the same (around 35% underestimation) over the years and were similar to the dual PEMS. This difference is still well within the permissible tolerance from the regulation (50%). We argued that the reason is the calibration material used by the manufacturer (spark discharge graphite). We demonstrated that calibrating with combustion soot could reduce the differences. The 10 nm PEMS gave similar results but with much smaller differences, indicating that the calibration material is of less importance for the Euro 7 step. The results showed that the measurement uncertainty has not increased but rather decreased for the specific PEMS switching from 23 nm to 10 nm.

## 1. Introduction

Air pollution was the second risk factor, after high blood pressure, for early death worldwide [1]. Particulate matter (PM) contributed 90% to air pollution disease burden or accounted for 7.8 million deaths. In addition to the health effects, PM is a concern due to its environmental impacts as well [2,3,4]. In Europe, around 250,000 premature deaths were estimated due to exposure to high PM [5]. There has been a significant decrease since 2010 [6], which, to a large degree, can be attributed to the reduction in vehicles’ exhaust PM. The most important change was the introduction of particulate filters to diesel-fueled vehicles. This was practically necessary because of the introduction of a stringent particle number (PN) limit in the European regulation in 2011 (Euro 5b step). Vehicle exhaust PM, typically called soot, consists of fractal-like aggregates with a mean diameter of 40–80 nm [7]. The primary particles (or spherules) are 10–30 nm in size [8,9,10].

The PN methodology in the regulation requires the measurement of solid particles with sizes larger than approximately 23 nm [11]. The aerosol is thermally treated and diluted, and then a device with an efficiency of around 50% at 23 nm counts the particles. Due to the steep counting efficiency requirements, practically all counting devices are condensation particle counters (CPCs): they grow the particles with saturated alcohol (typically butanol) vapor to sizes that can be detected by their optics. In 2017, with Euro 6c, the same PN limit was extended to all vehicles with direct fuel injection, not only on the chassis dynamometers but also on-road under real driving conditions. The Euro 6c step practically necessitated particulate filters for all vehicles with direct injection engines [12]. The on-road measurements are conducted with portable emission measurement systems (PEMSs). The PEMSs specifications are more relaxed compared to the laboratory-grade systems (LABSs) as they must be light, small, and robust [13]. Thus, instead of CPCs, some systems use diffusion chargers (DCs). DCs consist of a charger and an electrometer. Typically, a unipolar corona charger produces ions that charge particles. The resulting electric current of the charged particles is measured by a Faraday cup electrometer. An electrostatic field in between (“trap”) captures excess ions and small particles. The DCs usually keep the corona and electrostatic field voltages constant, but some models switch them on and off (modulated DCs) [14]. Internal functions convert the current to PN [15]. Systems of this principle of operation have a higher measurement uncertainty due to the dependency of the signal on the particle size. Furthermore, they have been found to be influenced by the particle structure [16,17]. On the other hand, CPCs have a dependency on the material affinity to the condensing liquid (butanol or isopropanol for on-road systems) [18,19]. The additional measurement uncertainty of PEMSs compared to LABSs is taken into account in the emission calculations with a so-called margin, which was 50% for PN until Euro 6d and was decreased to 34% with the Euro 6e step in 2023 [20,21].

Vehicles with port fuel injection do not have any PN (or mass) limit. However, some studies highlighted that they can emit high PN concentrations, in particular in the sub-23 nm range [12]. The “Particle Measurement Programme” (PMP) group, which also introduced the regulatory PN methodology [22], determined the technical specifications for systems measuring below 23 nm. The lower size was proposed to be approximately 10 nm, as lower sizes would result in very high measurement uncertainties due to the high particle losses in the setup and instruments. For LABSs, the technical specifications defined by PMP were added in the global technical regulation (GTR 15), which includes both 23 and 10 nm options [23]. For PEMSs, the PMP group presented the suggested technical specifications, but no regulation includes them currently. Some studies presented measurement uncertainties of the 10 nm PEMSs [20,24]. Nevertheless, discussions are still ongoing regarding the applicability of the 23 nm systems’ margin to the 10 nm systems.

One other topic of discussion is the calibration material. For LABSs, the thermal treatment unit and the CPCs are calibrated separately [11]. Any material can be used to calibrate the thermal treatment unit as long as it is thermally stable. For the 23 nm and 10 nm CPCs, the calibration material has to be emery oil or soot-like. For PEMSs, the material has to be soot-like. Soot-like particles include diffusion flame combustion soot and graphite particles produced by the spark discharge of graphite electrodes.

Euro 7 Regulation (EU) 2024/1257 entered into force in May 2024 [25]. The implementing acts (i.e., the technical details) will be adopted in May 2025. The application dates of new types of light-duty vehicles will be at the end of 2026, and for all vehicles before the end of 2027. For the exhaust emissions part, there are no major changes compared to Euro 6e [21], with one exception: the lower size of the particle number measurements decreased from 23 nm to 10 nm and the PN limit will be applicable to all vehicles, not only to those with direct fuel injection. Thus, it is of high interest to assess how commercial 10 nm systems, compliant to the PMP technical requirements, perform in the field, and most importantly how they compare with the well-established 23 nm systems, in particular the DC-based systems, which are supposed to have higher measurement uncertainty.

The aim of this paper is to assess a dual 23 nm and 10 nm system based on DC. Thus, compared to previous studies [24,26,27,28] the relative changes in the measurement uncertainty when changing the lower size from 23 nm to 10 nm will be assessed. In parallel, the impact of the calibration material on the response of the instrument will be investigated.

## 2. Materials and Methods

In this section, the experimental setup is described along with some theoretical background of the key instruments.

### 2.1. Laboratory and Portable Systems

The PN systems, both laboratory (LABS) and portable (PEMS), typically consist of the following parts [11]:

- Sampling line (optional), typically heated at a temperature of 100 °C.
- Primary dilution unit, typically heated at a temperature of 150 °C.
- Catalytically active tube, called a catalytic stripper (CS), typically heated at a temperature of 350 °C, to remove volatile particles.
- Secondary dilution (optional).
- Particle number counter or detector. For LABSs, this is a condensation particle counter (CPC), while for portable systems, CPCs or DCs are used.

### 2.2. Theoretical Background of Diffusion Chargers

In a diffusion charger (DC), ions from a corona discharge attach to particles and an electrometer measures their current. The measured quantity is called the active surface area [29]. The active surface area measured by DCs is significantly lower than the geometric surface [30]. Theoretically, in the free molecular regime, the measured current is proportional to particle diameter squared (e.g., [31]), and in the continuum regime, proportional to particle diameter. In the transition regime (20–200 nm), the exponent is 1.1–1.4 for most commercial instruments [32]. The signal of the DC-based instrument can be influenced by many parameters [32]:

- Ion properties: For example, if alcohol and water vapor attach to the ions, their mass increases and their mobility reduces, with an impact of 10% on the theoretical average particle charge.
- Temperature: At higher temperatures the ion velocity increases, and larger particles can acquire more charge. For example, an increase of +10 °C can increase the average particle charge of 100 nm particles by 2%.
- Pressure: At lower pressures, the ion mobility increases and, consequently, the average charge acquired by particles increases. Compared to ambient pressure, 100 mbar lower pressure can result in a 3% higher average particle charge at 100 nm.
- Dielectric constant (relative permittivity) of particles: The dielectric properties have an effect on the image forces. For example, polystyrene latex particles have 15% less average charge at 100 nm than metal particles. Graphite, black carbon, and combustion soot have similar values, with graphite at the lower edge of combustion soot’s values [33,34,35].
- Morphology: Fractal particles acquire more charge than compact particles of the same mobility (see, e.g., [36,37,38,39]). Differences of 10–30% can be observed for 100 nm particles. Analysis indicated that the electrical capacitance of loose agglomerates is larger than that of spherical particles with the same mobility; therefore, loose agglomerates can gain more charges [16].
- Pre-existing charge: If particles are already charged with the same polarity as the ions, the final charging level can be higher (see, e.g., [40,41]).
- Particle concentration: If the particle concentration entering the charger is high compared to the ion concentration, ion depletion leads to a lower average charge per particle.

### 2.3. PEMS Description

The PEMS of this study (MOVE from AVL, Graz, Austria) consisted of a hot diluter > 150 °C, an evaporation tube, a catalytic stripper at 300 °C, and a secondary diluter at 60 °C. The particle detector (Automotive Partector from Naneos, Windisch, Switzerland) [42,43,44,45] was a DC with a unipolar corona charger. A fraction of the charged particles was periodically removed by a pulsed electric field. The space charge change was determined in a Faraday cage. The user could set a 23 nm or 10 nm lower detection size in the software. This was achieved internally by modifying the pulsing electric field. This was the light-duty vehicle version without any neutralization technology upstream of the corona charger to remove all charged particles (typically urea particles) [41].

### 2.4. Experimental Setup

The assessment was conducted following various setups (Table 1):

1. Monodisperse calibration with laboratory soot-like aerosol vs. reference CPC, according to the calibration requirements described in Regulation (EU) 2017/1151 [46].
2. Polydisperse checks vs. reference CPC and reference LABS using laboratory soot-like aerosol. This is a relatively fast check as a quality check, but it is not described in the regulation [47].
3. Verifications vs. LABS at the tailpipe using vehicle exhaust. This check is not prescribed in the regulations, because LABSs are not allowed to be used at the tailpipe for light-duty applications. Nevertheless, the possibility to use LABSs at the tailpipe was developed for future introduction in the heavy-duty regulation and is described in a technical resolution [48]. It is common practice in engine laboratories [26,27,28].
4. Validations vs. LABSs at the dilution tunnel using vehicle exhaust. The procedure is described in the regulation [46] as a check of the proper operation of the PEMS.

**Figure 1:**
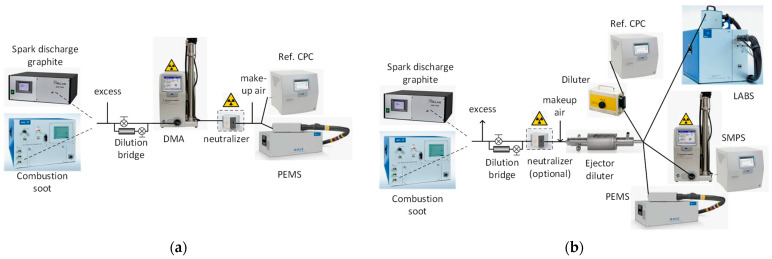
Experimental setups with laboratory aerosol: (**a**) monodisperse calibration; (**b**) polydisperse checks. CPC = condensation particle counter, DMA = differential mobility analyzer; LABS = laboratory-grade system; PEMS = portable emissions measurement system; SMPS = scanning mobility particle sizer (=DMA + CPC).

The description of the setup and the equipment follows. Figure 1a presents the calibration setup for monodisperse calibration. Two particle generators were used to produce soot-like aerosol:

- AVL particle generator (APG) from AVL, which includes a diffusion flame (mini CAST) generator (model 6.203 from Jing Ltd., Zollikofen, Switzerland) [49]. More specifically, the (mini) CAST generator produces particles by propane and air co-flow diffusion flame. The flame is quenched by nitrogen to keep the particle concentration high. Dilution stages reduce the particle concentration to the desired levels, while an evaporation tube at 350 °C removes volatiles.
- High-voltage spark discharge graphite electrode generator (DNP 3000 from PALAS, Karlsruhe, Germany) [50]. More specifically, due to the high-voltage spark discharge between the two graphite electrodes, the graphite material is evaporated [51]. Nitrogen transfers the vapors, which start to nucleate and coagulate. Dilution is used to reduce the particle concentration to the desired levels.

Both generators produce fractal-like particles with variable sizes. The primary particles of the diffusion flame generators are in the range 10–35 nm [52,53,54], while spark generators produce primary particles with sizes < 10 nm [55,56]. It should also be emphasized that spark discharge particles are already highly charged, as they acquire their charge in the spark region from the ions produced by the spark or by photoemission [57]. More info about the nanoparticles they produce can be found in the literature for spark discharge generators [55,56,58,59] and CAST [52,53,60,61].

The particle number concentration was adjusted in some tests by a dilution bridge. For the monodisperse tests, a differential mobility analyzer (DMA) [62] with a model 3080 long column (from TSI, Shoreview, MN, USA) and with a TSI 3077A ^85^Kr neutralizer (370 MBq, beta) was used to separate particles of a specific electrical mobility size (typically 15, 23, 30, 41, 50, 70, 100, and 200 nm). The estimated activity of our 12 year-old neutralizer was >45%. Neutralizers contain a radioactive source and, with diffusion charging, achieve an equilibrium particle charge distribution [63,64,65]. At a specific selected DMA size, it is possible that larger particles with more than one charge, but with the same electrical mobility with the singly charged particles at the selected size, exit the DMA. These larger and multiply charged particles will influence the calibration result. Carefully selecting the generated size distribution with the peak of the concentration below the selected size significantly reduces the concentration of multiply charged particles. In our experiments, the doubly charged particles were <5%, except at 200 nm (9.6%).

Particles at the exit of DMA are positively charged particles (negative voltage is applied to the DMA center electrode), and this could affect the charging efficiencies of the PEMS. For this reason, downstream of the DMA, we used a neutralizer to “neutralize” the aerosol (achieve an equilibrium charge distribution) (Grimm 5522, ^241^Am, 3.7 MBq, alpha with estimated activity >96% for its age of 23 years). In the past, we measured 200–350 fA downstream of the DMA for concentrations of 10,000–25,000 particles/cm^3^ without a neutralizer but only 60–70 fA with an additional neutralizer. The escaping ions from the neutralizer also result in some current (60–70 fA), and for this reason, it was not zero. Upstream of the instruments, high-efficiency particle air (HEPA)-filtered make-up flow air was added. Finally, a TSI 3752 CPC (cut-off 4 nm) was used as a reference instrument to check the absolute levels of particle number concentration. The reference CPC was fulfilling the requirements for calibration of both the 23 nm and 10 nm PEMSs. The monodisperse efficiency *E* of the PEMS at a particle size *i* is defined as follows:

*E_i,PEMS_* = *C_i,PEMS_*/*C_i,REF_* (1)

where *C_i_* is the measured concentration at size *i*. The tests were conducted with both the 23 nm and 10 nm configurations of the PEMS. The PEMS calibration certificates by the instrument manufacturer included efficiencies following a similar approach, as it is prescribed in the regulation. The generator used by the manufacturer was a spark discharge graphite from PALAS, as was ours.

Figure 1b presents the setup for the polydisperse checks. The polydisperse aerosol from the particle generators was diluted in an ejector dilutor, which provided enough flow for all instruments connected: the reference CPC downstream of a bifurcated diluter (DDS 560 from TOPAS GmbH, Dresden, Germany) [66], a scanning mobility particle sizer (SMPS) [67], the PEMS, and a LABS with both 23 nm and 10 nm CPCs.

For the polydisperse checks, the setup was similar to the monodisperse one but without DMA. The additional SMPS, i.e., the combination of DMA and CPC with appropriate software, could measure size distributions from 6 to 225 nm (sheath flow 15 lpm and sample flow 1.5 lpm). The SMPS was used to provide the geometric mean diameter (GMD) of the size distribution and to cross-check the measured concentration. The diffusion flame, as well as vehicle exhaust soot, is bipolarly charged with an overall neutral charge [68], thus, no neutralizers were used. For the spark discharge generator, which produces charged aerosol [50,56,69], the ^241^Am neutralizer was used in order to neutralize the highly charged particles, but for some tests, it was removed in order to investigate the charge effect on the PEMS efficiencies.

The polydisperse efficiency *E* for a size distribution with geometric mean diameter *GMD* was calculated using the CPC concentration (corrected for dilution) as a reference concentration according to Equation (2). Additionally, the PEMS vs. the LABS concentrations with the same cut-off (23 nm or 10 nm) were compared. Their difference *D* is an indication of the expected differences for the vehicle tests, where no reference CPC is available.

*E_GMD,PEMS_* = *C_GMD,PEMS_*/*C_GMD,REF_* (2)

*D_GMD_* = *C_GMD,PEMS_*/*C_GMD,LABS_* − 1 (3)

Although the details are not given in the description above, attention was paid to having conductive tubing to minimize electrostatic particle losses, choosing appropriate tube lengths, and ensuring similar losses between the reference instruments and PEMS.

**Figure 2:**
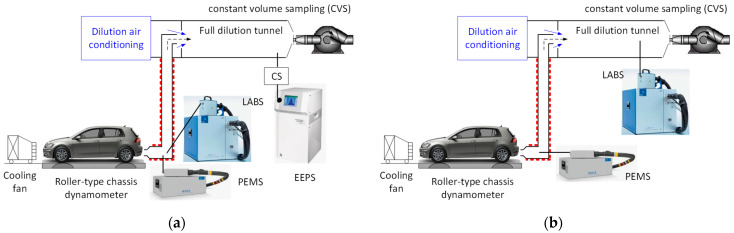
Experimental setups with polydisperse vehicle exhaust aerosol: (**a**) verification with laboratory system (LABS) at the tailpipe; (**b**) validation with LABS at the dilution tunnel with constant volume sampling (CVS). CS = catalytic stripper; EEPS = engine exhaust particle sizer; PEMS = portable emissions measurement system.

Figure 2a presents the setup using vehicle exhaust polydisperse aerosol. For the verification checks, the LABS (APC 489 from AVL) was connected to the tailpipe next to the PEMS. The LABS was connected to the tailpipe via a heated line (150 °C). At the full dilution tunnel, an engine exhaust particle sizer (EEPS) (from TSI) accompanied by a catalytic stripper measured solid particle size distributions. Two vehicles were used: a Euro 6d-temp diesel equipped with a diesel particulate filter (DPF) and a Euro 6b gasoline direct injection without a gasoline particulate filter (GPF). Note that the gasoline vehicle had a 6 × 10^12^ particles/km limit and the diesel 6 × 10^11^ particles/km.

Figure 2b presents the validation setup. The main difference with Figure 2a is that in the reference system, the advanced particle counter (APC 489, from AVL) was sampling from the dilution tunnel with a constant volume sampler (CVS). These tests are described in the regulation as a PEMS quality check, and for this reason, we had many tests available with three DC-based PEMSs at 23 nm. A total of 64 tests were conducted in three laboratories with 26 vehicles in the years 2021–2024.

## 3. Results

### 3.1. Laboratory Soot-like Aerosols

**Figure 3:**
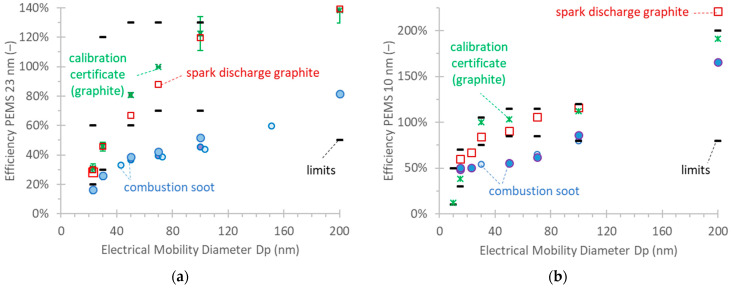
Monodisperse calibration of dual PEMS (MOVE from AVL) according to Figure 1a. Different symbol sizes indicate different repetitions on different days. The black lines give the limits from the regulation: (**a**) 23 nm PEMS. Asterisks are the calibration certificate values and error bars give one standard deviation of the last five calibration certificates. (**b**) 10 nm PEMS. Asterisks are the calibration certificate values of one calibration certificate.

Figure 3a presents the 23 nm PEMS efficiencies (Equation (1)) using monodisperse spark discharge graphite particles or diffusion flame combustion soot. Note that the *x*-axis is the (electrical) mobility diameter. The efficiencies of the last five calibration certificates are also plotted. There is a good agreement between the calibration certificates (using spark discharge graphite) and our graphite results. The graphite results are within the limits required by the legislation (small black lines). The combustion soot efficiencies are much lower.

Figure 3b plots the monodisperse calibration results for the 10 nm PEMS with the two soot-like aerosols, along with the PMP recommended future limits. There was only one calibration certificate available. The graphite is within the limits, with one exception at 200 nm. This point may be higher due to the existence of multiply charged particles that were not corrected. The combustion soot efficiencies are lower and, in most cases, below the limits.

**Figure 4:**
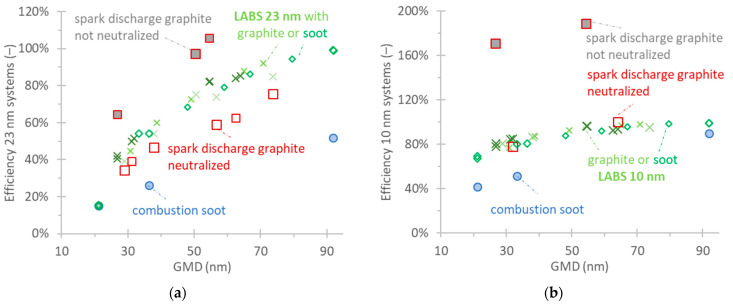
Polydisperse efficiencies of dual PEMS (MOVE from AVL) and LABS (APC 489 from AVL). Setup in Figure 1b: (**a**) 23 nm systems; (**b**) 10 nm systems. Note that the *x*-axis is the geometric mean diameter (GMD). The standard deviation was around 1.65. Squares (graphite) and circles (soot) are the PEMSs; green X (graphite) and diamonds (soot) are the LABSs. Different symbol sizes indicate different repetitions on different days.

The checks with polydisperse soot-like aerosols are plotted in Figure 4a for the 23 nm PEMS and Figure 4b for the 10 nm PEMS (efficiencies, Equation (2)). The figures also plot the efficiencies of the LABS. Note that the *x*-axis is geometric mean diameter (GMD). The geometric standard deviations were between 1.55 and 1.75.

The efficiencies with the combustion soot are very low, reaching only 50% at a large GMD of 91 nm. The efficiencies with the graphite are higher but still lower than those of the LABS. When the spark discharge aerosol was not neutralized, the efficiencies were much higher. The material (combustion soot or spark discharge graphite) did not impact the efficiencies of the LABS. The results were similar with the 10 nm PEMS. The agreement with the neutralized spark discharge graphite was close to the LABS. The non-neutralized aerosol, though, had a strong impact on the efficiencies.

**Figure 5:**
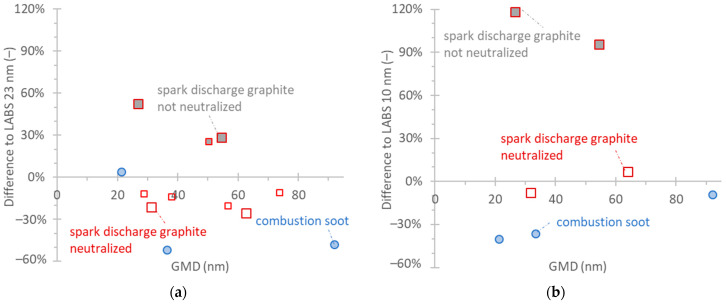
Comparison of PEMS with LABS measuring polydisperse soot-like aerosol (differences according to Equation (3)). Setup in Figure 1b: (**a**) 23 nm PEMS; (**b**) 10 nm PEMS. Different symbol sizes indicate different repetitions on different days.

The results of Figure 4 are also plotted in Figure 5, considering the respective LABS (i.e., the differences to the LABS system, Equation (3)) as a reference instrument. Such comparisons give a better indication of the differences that would be expected when measuring vehicle exhaust aerosol, where no reference CPC is available but only the LABS. For the 23 nm PEMS, the differences were on average −20% for neutralized spark discharge graphite but −50% for combustion soot. For the 10 nm PEMS, the differences were 0% for neutralized spark discharge graphite and −40% to −10% for combustion soot.

### 3.2. Vehicle Exhaust

**Figure 6:**
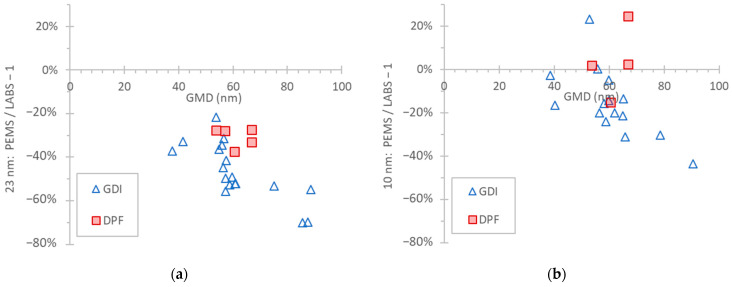
Comparison of PEMS with LABS, both at the tailpipe, measuring the exhaust of one DPF equipped and one GDI vehicle. Setup in Figure 2a: (**a**) 23 nm systems; (**b**) 10 nm systems.

The previous results were obtained with laboratory soot-like aerosol. However, it was not possible to determine which particle generator and kind of soot-like aerosol better simulates vehicle exhaust for PEMS calibrations. Figure 6 presents the differences between the PEMS and LABS, both connected to the tailpipe, when measuring vehicle exhaust aerosol from a gasoline-fueled vehicle with direct injection (GDI) and a diesel-fueled vehicle equipped with a diesel particulate filter (DPF). Each point is a test cycle (transient or at constant speed). The geometric mean diameter (GMD) was estimated from the instrument at the dilution tunnel (EEPS at CVS, Figure 2a). The differences spanned from −70% to −20% for the 23 nm PEMS and from −40% to +20% for the 10 nm PEMS.

**Figure 7:**
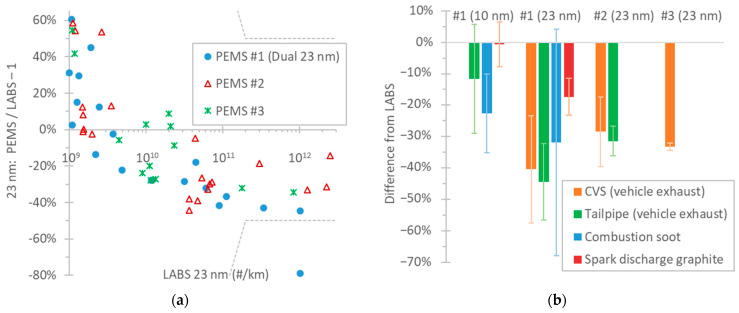
Comparison of three PEMSs with LABSs: (**a**) The 23 nm PEMSs were connected to the tailpipe and the 23 nm LABSs were connected to the dilution tunnel with constant volume sampling (CVS). Measurement over the years 2021 to 2024 with various vehicle technologies. Each point is a test cycle. Dotted lines indicate Euro 6d permissible tolerance. Setup in Figure 2b; (**b**) Summary of all measurements. Error bars give one standard deviation.

Figure 7a plots the validation tests with three 23 nm PEMSs over the years 2021 to 2024. The 23 nm LABSs were connected at the dilution tunnel, while the PEMSs were connected at the tailpipe, as prescribed in the regulation [46] (setup in Figure 2b).

The differences of PEMSs and LABSs were consistent: around −20% to −40% at emissions above 3 × 10^10^ particles/km. At lower emission levels, the differences became positive and increased as the PEMSs had reached their detection limit. Only in one case was the difference outside of the 50% or 1 × 10^11^ particles/km allowed tolerance prescribed in the regulation (−79%). The Euro 6e regulation decreased the tolerance to 42% or 8 × 10^10^ particles/km, whichever is larger. With the new tolerance, two additional points would fail the validation test (differences −44.6% and −42.8%). In all three cases, DPF regeneration took place and the PEMS was saturated (i.e., reached its maximum 2.5 × 10^7^ particles/cm^3^), partly explaining the underestimation. The differences of the three PEMSs were consistent, indicating that there was nothing particularly different with the dual PEMS.

Figure 7b summarizes the differences of the three PEMSs compared to LABSs with different aerosol sources, presented in the previous figures. The mean differences of the validation tests (all three 23 nm PEMSs at the tailpipe vs. LABSs at the CVS) were −28% to −40% for emission levels > 3 × 10^10^ particles/km (results presented in Figure 7a). The mean differences of the two 23 nm PEMSs vs. LABSs, both connected to the tailpipe, were −33% and −41% (results presented in Figure 6a). The 10 nm PEMS had a −12% difference compared to the 10 nm LABS (results presented in Figure 6b). Using laboratory aerosol, the 23 nm PEMS had a −32% difference with combustion soot and −17% with spark discharge graphite (results presented in Figure 5a). The 10 nm PEMS had a −23% difference with combustion soot and −1% with spark discharge graphite (results presented in Figure 5b). The comparisons covered a GMD range of 20 nm to 90 nm for soot and 25 nm to 75 nm for graphite.

## 4. Discussion

Euro 7 regulation (EU) 2024/1257 [25] reduced the lower detection size of the particle number (PN) systems from 23 nm to 10 nm and extended the limit to all vehicles (not only direct injection ones). The instruments are calibrated with monodisperse soot-like aerosol. The most commonly used generators are the diffusion flame combustion aerosol standard (CAST) and spark discharge based. The second one produces highly charged aerosols. There are discussions ongoing whether the measurement uncertainty of the instruments has remained the same or increased, especially for portable emissions measurement systems (PEMSs). There is particular interest for those instruments that do not optically count the particles, as conducted with condensation particle counters (CPCs), but charge them and measure their current, i.e., diffusion chargers (DCs). Due to their dependency on the size of measured particles, they can have higher measurement uncertainty; of course, other parameters play a role, such as flow accuracy and internal losses. Recently, a commercial DC-based system added the possibility of changing the lower detectable size from 23 nm to 10 nm by the user. This modification has the advantage that the instrument can be used with different lower detection sizes depending on the research purposes and/or the origin of the vehicle. Currently, the 10 nm lower size will be applied only in Europe at the end of 2026. In this study, we investigated this dual system with procedures following the regulation or variations to assess whether the measurement uncertainty levels remained the same. In parallel, we investigated the impact of the calibration material on the results.

One of the key findings was that the calibration material plays an important role for DC-based systems. The diffusion flame soot (monodisperse) efficiencies were almost half (45% lower) of the spark discharge graphite efficiencies for the 23 nm PEMS. Our calibration results with graphite were in very good agreement with the manufacturer’s calibration data, which were also based on graphite particles. The differences with monodisperse efficiencies were also reflected with polydisperse aerosol. For example, the efficiencies at around 37 nm (geometric mean diameter) were almost half (45% lower) of the spark discharge graphite efficiencies. The differences were even higher when the graphite particles were not neutralized. This is well known, and many studies with DC-based instruments have found impacts of the pre-existing charge on the response of the instrument [14,40,41]. Pre-existing charge results in a higher average charge per particle (compared to calibration) and consequently higher instrument response for the same particle number concentration. The sensitivity of the specific instrument to pre-charged particles was demonstrated also in the past with laboratory aerosol or with urea charged particles [41].

What is more interesting, though, is the high difference in the neutralized graphite particles with the diffusion flame soot. The PEMS keeps most of the parameters that can affect its response relatively constant or under control (see Section 2.2). For example, the sample humidity is kept low with the hot dilution in the instrument, the temperature of the particle detector is constant, and the ion concentration of the corona charger is enough for the concentrations at which the instrument can be challenged. In our tests, the ambient pressure was also relatively constant. One may argue that the spark discharge graphite particles were not neutralized adequately. However, the manufacturer used a tandem size classification setup and a neutralizer, and we used a neutralizer, thus, these results indicate the maximum neutralization that can be achieved with the current setups. Another reason for the differences may be the different morphology and fractality of the particles. It is well established that fractal particles are more efficiently charged than compact spherical/cubic particles for the same mobility diameter [36,37,70]. DCs measure the particles’ charge which depends on the surface area that is available for transport phenomena between particles and gas, the so-called active surface area [71]. The active surface area can be calculated by the mobility size of particles [72], with good agreement between direct active surface measurements and active surface calculation via the mobility size [73]. Different studies proposed that particles with the same mobility size but different morphology have different active surface area [74] and thus different DC responses [17,75]. Even if both diffusion flame and spark discharge fractal-like particles are formed via diffusion-limited cluster aggregation and particles have similar morphology [76], it cannot be excluded that different primary particle sizes produced by the two methods may result in morphological differences. Indeed, the primary spark discharge spherules during generation are typically small (<10 nm) [77], smaller than those from combustion soot (25 nm) [54,60]. Note that the dependency of DCs on particle morphology is impacted also by the design of the DC and can vary significantly [17,75]. Different responses of DCs for spark discharge graphite (neutralized or not) and combustion soot particles have also been observed before [17].

The 23 nm PEMS was compared to a laboratory-grade system (LABS) with combustion soot, graphite, and vehicle exhaust particles. The difference was around −15% with graphite particles, −35% with diffusion flame soot, and −35% to −45% with vehicle exhaust (26 tests with 14 vehicles). The differences with vehicle exhaust were higher than typically measured [24,27,28] but well within the permissible tolerance of the regulation (50% until Euro 6d or 42% from Euro 6e). Tests with the other two DC-based PEMSs also gave differences of −35% for 15 vehicles (39 tests) over three years. These results indicate that the diffusion flame soot results are closer to the real vehicle exhaust and should be preferable. Similar conclusions were drawn for DC-based equipment for the periodical technical inspection (PTI) of vehicles [17].

For the 10 nm PEMS, the results were quite similar. The graphite monodisperse efficiencies were within the proposed limits, while with combustion soot, they were lower (around 25 to 40% lower). Similarly, the combustion soot efficiency with polydisperse aerosol with a geometric mean diameter of 33 nm was 35% lower than graphite. Compared to a laboratory-grade system, the PEMS measured similar levels with graphite particles but 20% less with combustion soot and 10% less with vehicle exhaust. The non-neutralized graphite had a strong impact on the efficiencies, resulting in almost a 100% overestimation of the levels. The advantage of combustion soot as a calibration material was not as evident for the 10 nm PEMS, as the differences were much smaller. The results also support that changing the lower detection size to 10 nm does not increase the measurement uncertainty but rather decreases it, as deviations between instruments and/or calibration materials became smaller for the specific PEMS. The findings of this study were communicated to the instrument manufacturer and need further investigation with more combustion generators.

## 5. Conclusions

In this study, we assessed a portable emissions measurement system (PEMS) that can easily switch its lower size from 23 nm to 10 nm. We followed the (monodisperse) calibration procedures as prescribed in the regulation and compared our results with the manufacturers’ results using two aerosols: diffusion flame combustion soot and spark discharge graphite. We also checked the efficiency of the instrument (i.e., response compared to the reference instrument) with polydisperse aerosol. We finally compared it with laboratory-grade instruments using the two aerosols and vehicle exhaust.

Our results were in good agreement with the manufacturer’s calibration certificate with monodisperse spark discharge graphite particles. However, the response of the 23 nm instrument to monodisperse and/or polydisperse combustion soot was much lower (−35%); although, it was still well within the permissible tolerance from the regulation (50%). Such underestimation was also noticed with vehicle exhaust. Other validation tests with two more PEMSs confirmed the findings. Thus, our results suggest that diffusion flame combustion soot is closer to vehicle exhaust and should be preferred as a calibration material. The results with the 10 nm PEMS were similar, with nevertheless much smaller deviations between calibration materials and/or the two reference instruments. More studies with different soot generators should confirm these findings.

## Figures and Tables

**Table 1:** Overview of the tests conducted. # refers to the code of PEMS.

Test	Calibration Aerosol	Ref. Instruments	PEMS	Setup
Monodisperse calibration	Graphite or combustion soot	Ref. CPC	#1	Figure 1a
Polydisperse checks	Graphite or combustion soot	Ref. CPC, SMPS, LABS	#1	Figure 1b
Verifications	Vehicle exhaust (1 × DPF, 1 × GDI)	LABS at tailpipe	#1, #2	Figure 2a
Validations	Vehicle exhaust (many)	LABS at CVS	#1, #2, #3	Figure 2b

CPC = condensation particle counter; CVS = constant volume sampler; DPF = diesel particulate filter; GDI = gasoline direct injection; LABS = laboratory-grade system; SMPS = scanning mobility particle sizer.

## Data Availability

Data are available from the corresponding author upon request.

## Abbreviations

APC	advanced particle counter
APG	AVL particle generator
CAST	combustion aerosol standard
CPC	condensation particle counter
CS	catalytic stripper
CVS	constant volume sampler (dilution tunnel)
DC	diffusion charger
DMA	differential mobility analyzer
DPF	diesel particulate filter
EEPS	engine exhaust particle sizer
EU	European Union
GDI	gasoline direct injection
GMD	geometric mean diameter
GPF	gasoline particulate filter
GTR	global technical regulation
HEPA	high-efficiency particle air (filter)
JRC	Joint Research Centre
LABS	laboratory-grade system
PEMS	portable emission measurement system
PM	particulate matter
PMP	particle measurement programme
PN	particle number
PTI	periodical technical inspection
SMPS	scanning mobility particle sizer
TP	tailpipe

## References

[1] Health Effects Institute (2024). State of Global Air 2024.

[2] Manisalidis I., Stavropoulou E., Stavropoulos A., Bezirtzoglou E. (2020). Environmental and Health Impacts of Air Pollution: A Review. Front. Public Health.

[3] Aslam I., Roeffaers M.B.J. (2022). Carbonaceous Nanoparticle Air Pollution: Toxicity and Detection in Biological Samples. Nanomaterials.

[4] Chauhan B.V.S., Corada K., Young C., Smallbone K.L., Wyche K.P. (2024). Review on Sampling Methods and Health Impacts of Fine (PM2.5, ≤2.5 μm) and Ultrafine (UFP, PM0.1, ≤0.1 μm) Particles. Atmosphere.

[5] European Environmental Agency (EEA) (2022). Air Quality in Europe 2022.

[6] European Environmental Agency (EEA) (2023). Premature Deaths Due to Exposure to Fine Particulate Matter in Europe. https://www.eea.europa.eu/en/analysis/indicators/health-impacts-of-exposure-to.

[7] Harris S.J., Maricq M.M. (2001). Signature Size Distributions for Diesel and Gasoline Engine Exhaust Particulate Matter. J. Aerosol Sci..

[8] Müller J.-O., Su D.S., Jentoft R.E., Wild U., Schlögl R. (2006). Diesel Engine Exhaust Emission: Oxidative Behavior and Microstructure of Black Smoke Soot Particulate. Environ. Sci. Technol..

[9] Barone T.L., Storey J.M.E., Youngquist A.D., Szybist J.P. (2012). An Analysis of Direct-Injection Spark-Ignition (DISI) Soot Morphology. Atmos. Environ..

[10] Liati A., Dimopoulos Eggenschwiler P., Schreiber D., Zelenay V., Ammann M. (2013). Variations in Diesel Soot Reactivity along the Exhaust After-Treatment System, Based on the Morphology and Nanostructure of Primary Soot Particles. Combust. Flame.

[11] Giechaskiel B., Melas A., Martini G., Dilara P. (2021). Overview of Vehicle Exhaust Particle Number Regulations. Processes.

[12] Giechaskiel B., Joshi A., Ntziachristos L., Dilara P. (2019). European Regulatory Framework and Particulate Matter Emissions of Gasoline Light-Duty Vehicles: A Review. Catalysts.

[13] Giechaskiel B., Bonnel P., Perujo A., Dilara P. (2019). Solid Particle Number (SPN) Portable Emissions Measurement Systems (PEMS) in the European Legislation: A Review. Int. J. Environ. Res. Public Health.

[14] Knoll M., Schriefl M.A., Nishida R.T., Bergmann A. (2021). Impact of Pre-Charged Particles on Steady State and Pulsed Modes of Unipolar Diffusion Chargers. Aerosol Sci. Technol..

[15] Schriefl M.A., Bergmann A., Fierz M. (2019). Design Principles for Sensing Particle Number Concentration and Mean Particle Size with Unipolar Diffusion Charging. IEEE Sens. J..

[16] Shin W.G., Wang J., Mertler M., Sachweh B., Fissan H., Pui D.Y.H. (2010). The Effect of Particle Morphology on Unipolar Diffusion Charging of Nanoparticle Agglomerates in the Transition Regime. J. Aerosol Sci..

[17] Vasilatou K., Wälchli C., Auderset K., Burtscher H., Hammer T., Giechaskiel B., Melas A. (2023). Effects of the Test Aerosol on the Performance of Periodic Technical Inspection Particle Counters. J. Aerosol Sci..

[18] Wang X., Caldow R., Sem G.J., Hama N., Sakurai H. (2010). Evaluation of a Condensation Particle Counter for Vehicle Emission Measurement: Experimental Procedure and Effects of Calibration Aerosol Material. J. Aerosol Sci..

[19] Giechaskiel B., Melas A. (2022). Impact of Material on Response and Calibration of Particle Number Systems. Atmosphere.

[20] Giechaskiel B., Lähde T., Melas A.D., Valverde V., Clairotte M. (2021). Uncertainty of Laboratory and Portable Solid Particle Number Systems for Regulatory Measurements of Vehicle Emissions. Environ. Res..

[21] European Commission Commission Regulation (EU) (2023). 2023/443 of 8 February 2023 Amending Regulation (EU) 2017/1151 as Regards the Emission Type Approval Procedures for Light Passenger and Commercial Vehicles. Off. J. Eur. Union.

[22] Giechaskiel B., Mamakos A., Andersson J., Dilara P., Martini G., Schindler W., Bergmann A. (2012). Measurement of Automotive Nonvolatile Particle Number Emissions within the European Legislative Framework: A Review. Aerosol Sci. Technol..

[23] GTR Global Technical Regulation 15. https://unece.org/transport/standards/transport/vehicle-regulations-wp29/global-technical-regulations-gtrs.

[24] Giechaskiel B., Lähde T., Gandi S., Keller S., Kreutziger P., Mamakos A. (2020). Assessment of 10-nm Particle Number (PN) Portable Emissions Measurement Systems (PEMS) for Future Regulations. Int. J. Environ. Res. Public Health.

[25] European Commission Regulation (EU) (2024). 2024/1257 of the European Parliament and of the Council of 24 April 2024 on Type-Approval of Motor Vehicles and Engines and of Systems, Components and Separate Technical Units Intended for Such Vehicles, with Respect to Their Emissions and Battery Durability (Euro 7), Amending Regulation (EU) 2018/858 of the European Parliament and of the Council and Repealing Regulations (EC) No 715/2007 and (EC) No 595/2009 of the European Parliament and of the Council, Commission Regulation (EU) No 582/2011, Commission Regulation (EU) 2017/1151, Commission Regulation (EU) 2017/2400 and Commission Implementing Regulation (EU) 2022/1362. Text with EEA Relevance. Off. J. Eur. Union.

[26] Giechaskiel B., Schwelberger M., Delacroix C., Marchetti M., Feijen M., Prieger K., Andersson S., Karlsson H.L. (2018). Experimental Assessment of Solid Particle Number Portable Emissions Measurement Systems (PEMS) for Heavy-Duty Vehicles Applications. J. Aerosol Sci..

[27] Khan M.Y., Patel M., Scott N., Liew C.M., Peng C., Luo W., Rahman M., Gramlich N., Eames J., Phillips J.A. (2020). Assessment of In-Use Solid Particle Number Measurement Systems against Laboratory Systems.

[28] Khan M.Y., Agarwal N., Panda S., Desai A.T., Wilkinson J.C., Chaille E., Vats S., Salemme T.L., Ragupathy T. (2024). Assessment of Condensation Particle Counter-Based Portable Solid Particle Number System for Applications with High Water Content in Exhaust.

[29] Siegmann K., Siegmann H.C. (2000). Fast and Reliable “In Situ” Evaluation of Particles and Their Surfaces with Special Reference to Diesel Exhaust.

[30] Ku B.K., Kulkarni P. (2012). Comparison of Diffusion Charging and Mobility-Based Methods for Measurement of Aerosol Agglomerate Surface Area. J. Aerosol Sci..

[31] Ku B.K., Maynard A.D. (2005). Comparing Aerosol Surface-Area Measurements of Monodisperse Ultrafine Silver Agglomerates by Mobility Analysis, Transmission Electron Microscopy and Diffusion Charging. J. Aerosol Sci..

[32] Dhaniyala S., Fierz M., Keskinen J., Marjamäki M., Kulkarni P., Baron P.A., Willeke K. (2011). Instruments Based on Electrical Detection of Aerosols. Aerosol Measurement: Principles, Techniques, and Applications.

[33] Ouf F.-X., Sillon P. (2009). Charging Efficiency of the Electrical Low Pressure Impactor’s Corona Charger: Influence of the Fractal Morphology of Nanoparticle Aggregates and Uncertainty Analysis of Experimental Results. Aerosol Sci. Technol..

[34] Gümüş E., Yağımlı M., Arca E. (2023). Investigation of the Dielectric Properties of Graphite and Carbon Black-Filled Composites as Electromagnetic Interference Shielding Coatings. Appl. Sci..

[35] Walter S., Schwanzer P., Hagen G., Rabl H.-P., Dietrich M., Moos R. (2023). Soot Monitoring of Gasoline Particulate Filters Using a Radio-Frequency-Based Sensor. Sensors.

[36] Oh H., Park H., Kim S. (2004). Effects of Particle Shape on the Unipolar Diffusion Charging of Nonspherical Particles. Aerosol Sci. Technol..

[37] Jung H., Kittelson D.B. (2005). Characterization of Aerosol Surface Instruments in Transition Regime. Aerosol Sci. Technol..

[38] Wang J., Shin W.G., Mertler M., Sachweh B., Fissan H., Pui D.Y.H. (2010). Measurement of Nanoparticle Agglomerates by Combined Measurement of Electrical Mobility and Unipolar Charging Properties. Aerosol Sci. Technol..

[39] Bełkowska-Wołoczko D. (2019). Introduction of Fractal-like Agglomerates to the Algorithm for Calculating Surface Area Concentrations of PM1. Air Qual. Atmos. Health.

[40] Qi C., Asbach C., Shin W.G., Fissan H., Pui D.Y.H. (2009). The Effect of Particle Pre-Existing Charge on Unipolar Charging and its Implication on Electrical Aerosol Measurements. Aerosol Sci. Technol..

[41] Schwelberger M., Mamakos A., Fierz M., Giechaskiel B. (2019). Experimental Assessment of an Electrofilter and a Tandem Positive-Negative Corona Charger for the Measurement of Charged Nanoparticles Formed in Selective Catalytic Reduction Systems. Appl. Sci..

[42] Fierz M., Meier D., Steigmeier P., Burtscher H. (2014). Aerosol Measurement by Induced Currents. Aerosol Sci. Technol..

[43] Todea A.M., Beckmann S., Kaminski H., Asbach C. (2015). Accuracy of Electrical Aerosol Sensors Measuring Lung Deposited Surface Area Concentrations. J. Aerosol Sci..

[44] Todea A.M., Beckmann S., Kaminski H., Bard D., Bau S., Clavaguera S., Dahmann D., Dozol H., Dziurowitz N., Elihn K. (2017). Inter-Comparison of Personal Monitors for Nanoparticles Exposure at Workplaces and in the Environment. Sci. Total Environ..

[45] Asbach C., Alexander C., Clavaguera S., Dahmann D., Dozol H., Faure B., Fierz M., Fontana L., Iavicoli I., Kaminski H. (2017). Review of Measurement Techniques and Methods for Assessing Personal Exposure to Airborne Nanomaterials in Workplaces. Sci. Total Environ..

[46] European Commission Commission Regulation (EU) (2017). 2017/1151 Supplementing Regulation (EC) No 715/2007 of the European Parliament and of the Council on Type-Approval of Motor Vehicles with Respect to Emissions from Light Passenger and Commercial Vehicles (Euro 5 and Euro 6) and on Access to Vehicle Repair and Maintenance Information, Amending Directive 2007/46/EC of the European Parliament and of the Council, Commission Regulation (EC) No 692/2008 and Commission Regulation (EU) No 1230/2012 and Repealing Commission Regulation (EC) No 692/2008. Off. J. Eur. Union.

[47] Giechaskiel B., Bergmann A. (2012). On-Site Checks of the Particle Number Measurement Systems with Polydisperse Aerosol. SAE Int. J. Engines.

[48] (2021). PMP GRPE 85-04: Proposed Amendments to ECE/TRANS/WP.29/GRPE/2021/17. https://unece.org/transport/events/wp29grpe-working-party-pollution-and-energy-85th-session.

[49] Giechaskiel B., Melas A. (2022). Comparison of Particle Sizers and Counters with Soot-like, Salt, and Silver Particles. Atmosphere.

[50] Schwyn S., Garwin E., Schmidt-Ott A. (1988). Aerosol Generation by Spark Discharge. J. Aerosol Sci..

[51] Horvath H., Gangl M. (2003). A Low-Voltage Spark Generator for Production of Carbon Particles. J. Aerosol Sci..

[52] Mamakos A., Khalek I., Giannelli R., Spears M. (2013). Characterization of Combustion Aerosol Produced by a Mini-CAST and Treated in a Catalytic Stripper. Aerosol Sci. Technol..

[53] Ess M.N., Vasilatou K. (2019). Characterization of a New miniCAST with Diffusion Flame and Premixed Flame Options: Generation of Particles with High EC Content in the Size Range 30 nm to 200 nm. Aerosol Sci. Technol..

[54] Haller T., Rentenberger C., Meyer J.C., Felgitsch L., Grothe H., Hitzenberger R. (2019). Structural Changes of CAST Soot during a Thermal–Optical Measurement Protocol. Atmos. Meas. Tech..

[55] Meuller B.O., Messing M.E., Engberg D.L.J., Jansson A.M., Johansson L.I.M., Norlén S.M., Tureson N., Deppert K. (2012). Review of Spark Discharge Generators for Production of Nanoparticle Aerosols. Aerosol Sci. Technol..

[56] Pfeiffer T.V., Feng J., Schmidt-Ott A. (2014). New Developments in Spark Production of Nanoparticles. Adv. Powder Technol..

[57] Maisser A., Barmpounis K., Attoui M.B., Biskos G., Schmidt-Ott A. (2015). Atomic Cluster Generation with an Atmospheric Pressure Spark Discharge Generator. Aerosol Sci. Technol..

[58] Kornyushin D., Musaev A., Patarashvili A., Buchnev A., Arsenov P., Ivanov M., Vershinina O., Kameneva E., Volkov I., Efimov A. (2023). Effect of the Gas Temperature on Agglomeration of Au Nanoparticles Synthesized by Spark Discharge and Their Application in Surface-Enhanced Raman Spectroscopy. Metals.

[59] Lizunova A.A., Borisov V.I., Malo D., Musaev A.G., Kameneva E.I., Efimov A.A., Volkov I.A., Buchnev A.I., Shuklov I.A., Ivanov V.V. (2023). Spark Discharge Synthesis and Characterization of Ge/Sn Janus Nanoparticles. Nanomaterials.

[60] Moore R.H., Ziemba L.D., Dutcher D., Beyersdorf A.J., Chan K., Crumeyrolle S., Raymond T.M., Thornhill K.L., Winstead E.L., Anderson B.E. (2014). Mapping the Operation of the Miniature Combustion Aerosol Standard (Mini-CAST) Soot Generator. Aerosol Sci. Technol..

[61] Kim J., Bauer H., Dobovičnik T., Hitzenberger R., Lottin D., Ferry D., Petzold A. (2015). Assessing Optical Properties and Refractive Index of Combustion Aerosol Particles Through Combined Experimental and Modeling Studies. Aerosol Sci. Technol..

[62] Liu B.Y.H., Pui D.Y.H. (1975). On the Performance of the Electrical Aerosol Analyzer. J. Aerosol Sci..

[63] Wiedensohler A. (1988). An Approximation of the Bipolar Charge Distribution for Particles in the Submicron Size Range. J. Aerosol Sci..

[64] Ji J.H., Bae G.N., Hwang J. (2004). Characteristics of Aerosol Charge Neutralizers for Highly Charged Particles. J. Aerosol Sci..

[65] Gopalakrishnan R., McMurry P.H., Hogan C.J. (2015). The Bipolar Diffusion Charging of Nanoparticles: A Review and Development of Approaches for Non-Spherical Particles. Aerosol Sci. Technol..

[66] Giechaskiel B., Melas A., Mamakos A. (2023). Assessment of Two Condensation Particle Counters (CPCs) in Photometric Mode for High Concentration Exhaust Emission Measurements. Combust. Engines.

[67] Wang S.C., Flagan R.C. (1990). Scanning Electrical Mobility Spectrometer. Aerosol Sci. Technol..

[68] Maricq M.M. (2006). On the Electrical Charge of Motor Vehicle Exhaust Particles. J. Aerosol Sci..

[69] Bau S., Witschger O., Gensdarmes F., Thomas D., Borra J.-P. (2010). Electrical Properties of Airborne Nanoparticles Produced by a Commercial Spark-Discharge Generator. J. Nanoparticle Res..

[70] Maricq M.M. (2013). Monitoring Motor Vehicle PM Emissions: An Evaluation of Three Portable Low-Cost Aerosol Instruments. Aerosol Sci. Technol..

[71] Keller A., Fierz M., Siegmann K., Siegmann H.C., Filippov A. (2001). Surface Science with Nanosized Particles in a Carrier Gas. J. Vac. Sci. Technol. A Vac. Surf. Film..

[72] Heitbrink W.A., Evans D.E., Ku B.K., Maynard A.D., Slavin T.J., Peters T.M. (2008). Relationships Among Particle Number, Surface Area, and Respirable Mass Concentrations in Automotive Engine Manufacturing. J. Occup. Environ. Hyg..

[73] Gini M.I., Helmis C., Melas A.D., Papanastasiou D., Orfanopoulos G., Giannakopoulos K.P., Drossinos Y., Eleftheriadis K. (2016). Characterization of Carbon Fractal-like Aggregates by Size Distribution Measurements and Theoretical Calculations. Aerosol Sci. Technol..

[74] Nishida R.T., Johnson T.J., Boies A.M., Hochgreb S. (2019). Measuring Aerosol Active Surface Area by Direct Ultraviolet Photoionization and Charge Capture in Continuous Flow. Aerosol Sci. Technol..

[75] Schriefl M.A., Nishida R.T., Knoll M., Boies A.M., Bergmann A. (2020). Characterization of Particle Number Counters Based on Pulsed-Mode Diffusion Charging. Aerosol Sci. Technol..

[76] Sorensen C.M. (2011). The Mobility of Fractal Aggregates: A Review. Aerosol Sci. Technol..

[77] Tabrizi N.S., Ullmann M., Vons V.A., Lafont U., Schmidt-Ott A. (2009). Generation of Nanoparticles by Spark Discharge. J. Nanoparticle Res..

